# rAAV2/7 vector-mediated overexpression of alpha-synuclein in mouse substantia nigra induces protein aggregation and progressive dose-dependent neurodegeneration

**DOI:** 10.1186/1750-1326-8-44

**Published:** 2013-11-25

**Authors:** Marusela Oliveras-Salvá, Anke Van der Perren, Nicolas Casadei, Stijn Stroobants, Silke Nuber, Rudi D’Hooge, Chris Van den Haute, Veerle Baekelandt

**Affiliations:** 1 Department of Neurosciences and Leuven Research Institute for Neuroscience and Disease (LIND), KU Leuven, Laboratory for Neurobiology and Gene Therapy, Kapucijnenvoer 33, box 7001, 3000 Leuven, Belgium; 2 Institute of Medical Genetics and Applied Genomics, University of Tübingen, Calwerstrasse 7, 72076 Tübingen, Germany; 3 Department of Psychology, KU Leuven, Laboratory of Biological Psychology, Tiensestraat 102, 3000, Leuven, Belgium; 4 Department of Neurosciences, Medical Teaching Facility, University of California San Diego, 9500 Gilman Dr, La Jolla, CA 92093 USA

**Keywords:** Parkinson’s disease, Alpha-synuclein, Mouse, Model, AAV, A53T, Neurodegeneration, Phosphorylation, Aggregation, Behaviour

## Abstract

**Background:**

Alpha-synuclein is a key protein implicated in the pathogenesis of Parkinson's disease (PD). It is the main component of the Lewy bodies, a cardinal neuropathological feature in the disease. In addition, whole locus multiplications and point mutations in the gene coding for alpha-synuclein lead to autosomal dominant monogenic PD. Over the past decade, research on PD has impelled the development of new animal models based on alpha-synuclein. In this context, transgenic mouse lines have failed to reproduce several hallmarks of PD, especially the strong and progressive dopaminergic neurodegeneration over time that occurs in the patients. In contrast, viral vector-based models in rats and non-human primates display prominent, although highly variable, nigral dopaminergic neuron loss. However, the few studies available on viral vector-mediated overexpression of alpha-synuclein in mice report a weak neurodegenerative process and no clear Lewy body-like pathology. To address this issue, we performed a comprehensive comparative study of alpha-synuclein overexpression by means of recombinant adeno-associated viral vectors serotype 2/7 (rAAV2/7) at different doses in adult mouse substantia nigra.

**Results:**

We noted a significant and dose-dependent alpha-synucleinopathy over time upon nigral viral vector-mediated alpha-synuclein overexpression. We obtained a strong, progressive and dose-dependent loss of dopaminergic neurons in the substantia nigra, reaching a maximum of 82% after 8 weeks. This effect correlated with a reduction in tyrosine hydroxylase immunoreactivity in the striatum. Moreover, behavioural analysis revealed significant motor impairments from 12 weeks after injection on. In addition, we detected the presence of alpha-synuclein-positive aggregates in the remaining surviving neurons. When comparing wild-type to mutant A53T alpha-synuclein at the same vector dose, both induced a similar degree of cell death. These data were supported by a biochemical analysis that showed a net increase in soluble and insoluble alpha-synuclein expression over time to the same extent for both alpha-synuclein variants.

**Conclusions:**

In conclusion, our *in vivo* data provide evidence that strong and significant alpha-synuclein-induced neuropathology and progressive dopaminergic neurodegeneration can be achieved in mouse brain by means of rAAV2/7.

## Background

Parkinson’s disease (PD) is a common neurodegenerative movement disorder that affects 1% of the population aged over 65. Clinically, PD is characterized by major motor symptoms consisting of bradykinesia, cogwheel rigidity, resting tremor and postural instability. The neuropathological cardinal features of the disease comprise both the preferential loss of the dopaminergic neurons of the substantia nigra (SN) and the prevalence of large cytoplasmic inclusions rich in alpha-synuclein (α-synuclein) protein, named Lewy bodies (LBs), which are present in the remaining cells [1]. In addition, whole locus duplications and triplications and genetic single mutations (A53T, A30P, E46K, H50Q, G51D) in the *SNCA* gene coding for α-synuclein result in autosomal-dominant familial forms of PD [2-8]. The severity of the phenotype correlates with the *SNCA* genomic dosage implying that high levels of α-synuclein can in fact trigger the neurodegenerative process [9]. Finally, genome-wide association studies also support this critical role for α-synuclein in the pathogenesis of PD [10,11]. The discovery of α-synuclein both as a genetic cause for the disease and as the major component of the LBs in sporadic and familial cases of PD has strengthened the link between sporadic and hereditary PD forms and the possibility of an underlying common mechanism at the origin of the disease.

Since α-synuclein is so tightly linked to the pathogenesis of PD, numerous transgenic mouse lines overexpressing wild-type (WT) or mutant α-synuclein were developed over the past decade, aiming at replicating the neuropathology seen in patients [12,13]. Despite the fact that these mice have proven useful in modelling some features of the disease such as the abnormal accumulation of α-synuclein in cells, they do not display a convincing progressive degeneration of the nigral dopaminergic neurons nor a dopamine-dependent motor phenotype. Conversely, the overexpression of α-synuclein by means of viral vectors in rodents and non-human primates induces a progressive dopaminergic cell loss over time as the most prominent feature (see [14] for a review). We have previously reported the overexpression of α-synuclein in mouse and rat brain using lentiviral vectors [15-17], but recombinant adeno-associated viral (rAAV) vectors have steadily gained more interest to express a gene of interest in the brain in a controlled spatio-temporal manner because of their high titers and high tropism for dopaminergic cells, especially for the newer serotypes [18-22].

Viral vector-based α-synuclein rat and non-human primate models display several cardinal neuropathological features of PD. Indeed, rAAV vector-mediated overexpression of either WT or mutant A53T α-synuclein induces a 20 to 80% dopaminergic cell loss in 3 to 8 weeks in rats, depending on the study and the serotype used [23-28], and a 30 to 60% dopaminergic neuron loss at 4 months after injection in marmosets [29]. Beside the dopaminergic cell death, rat and non-human primate models exhibit other hallmarks of PD such as the prevalence of α-synuclein-positive inclusions in the SN or the presence of a phosphorylated form of α-synuclein at serine 129 (P-S129), which is largely abundant in the LBs of PD patients [30,31]. Although promising, these viral vector-based α-synuclein models still suffer from a certain degree of variability and slow progression of the phenotype, hindering their value for testing novel therapeutics. To address this, we have taken a novel vector, rAAV2/7, which can be prepared at high titer and for which we have shown its efficient transduction of the dopaminergic neurons of the SN [32]. We have developed an improved rat model for PD by means of rAAV2/7 vector-mediated overexpression of A53T α-synuclein in the SN, which displays progressive and robust neurodegeneration (Van der Perren et al., submitted). Intriguingly, the few studies with α-synuclein rAAV vectors performed in mice until now report either no or very limited dopaminergic neuron loss, with a maximum of 20 to 35% at 2 to 6 months after injection [33-38]. Furthermore, no obvious α-synuclein-positive inclusions were detected in the surviving cell bodies [35].

In view of the increasing number of transgenic and knockout mice, the availability of a viral vector-based α-synuclein mouse model would offer several interesting scientific possibilities. Therefore, we decided to explore whether it is possible to obtain a robust parkinsonian phenotype in mice with α-synuclein-encoding rAAV vectors. We performed a comprehensive comparative study of 3 different vector doses of rAAV2/7-α-synuclein WT in mouse SN and analyzed them at separate time points up to 2 months after surgery. In addition, we compared the overexpression of human WT and mutant A53T α-synuclein in mouse SN delivered at the same vector dose. We assessed the changes induced by the sustained localized overexpression of α-synuclein in mouse SN by means of motor behavioural tests, immunohistochemical stainings followed by stereological quantifications, and protein solubility analysis. We succeeded in developing a mouse model based on rAAV2/7 vector-mediated overexpression of α-synuclein with robust dopaminergic cell death and α-synuclein inclusions in the SN.

## Methods

### rAAV vector design and production

The plasmids for the rAAV vector production were previously described [39]. These plasmids include the construct for the AAV2/7 serotype, the AAV transfer plasmid and the pAdvDeltaF6 adenoviral helper plasmid. The transfer plasmid encoded eGFP or the human WT or A53T α-synuclein transgene, followed by the woodchuck hepatitis posttranscriptional regulatory element, under the control of the CMVie-enhanced-synapsin1 promoter.

The viral vectors were produced at the Leuven Viral Vector Core (LVVC, http://www.kuleuven.be/molmed/research/research_viral_vector_technology.html) from the KU Leuven (Leuven, Belgium). Vector production and purification were performed as described in detail elsewhere [19]. The final titers for the concentrated vector stocks ranged between 8,0E + 11 and 2,7E + 12 genome copies per millilitre (GC/ml) as determined by quantitative PCR.

### Stereotactic surgery

Housing and handling of mice was done in compliance with the European Communities Council Directive of 24 November 1986 (86/609/EEC) and approved by the Bioethical Committee of the KU Leuven. All animals were housed under 12/12 h light/dark cycle with access to food and water *ad libitum*. Female, 8 weeks old, C57BL/6 mice received the human WT α-synuclein rAAV2/7 vector at different titers: 2,6E + 11 GC/ml; 4,0E + 11 GC/ml or 8, 0E + 11 GC/ml. The rAAV2/7-eGFP vector was injected at 4,0E + 11 GC/ml or 8,0E + 11 GC/ml. The human A53T α-synuclein rAAV2/7 vector was tested at a single dose: 4,0E + 11 GC/ml. The animals were anaesthetized and placed in a stereotactic head frame (Stoelting, IL, USA). After making a midline incision of the scalp, a burr hole was drilled in the appropriate location for the SN at the right site of the skull. 2 μl of viral vector were injected at a rate of 0.25 μl/min with a 30-gauge needle on a 10 μl Hamilton syringe. The needle was left in place for an additional 5 minutes before being slowly withdrawn from the brain. Coordinates for mouse SN were anteroposterior (AP) -3.1 mm and mediolateral (ML) -1.2 mm relative to bregma, and dorsoventral (DV) -4.0 mm from the dural surface [40].

### Motor behaviour

Three groups of mice (n = 10) were injected with rAAV2/7-eGFP (8,0E + 11 GC/ml) or human WT α-synuclein rAAV2/7 vector (8,0E + 11 GC/ml and 4,0E + 11 GC/ml) for behavioural evaluation in the cylinder test, the rotarod test and the open field test.

The cylinder test, which measures asymmetry in spontaneous forelimb use, was performed at 1, 4, 8 and 12 weeks after injection. Mice were placed individually inside a glass cylinder (12 cm diameter, 22 cm height) positioned in front of two vertical mirrors in order to be able to view the mouse from all angles. No habituation of the animals to the testing cylinder was allowed before video-recording. Video-recordings were examined by an observer blinded to the animal’s identity. Between 20 and 30 wall touches per animal (contacts with fully extended digits executed with the forelimb ipsilateral and contralateral to the lesion) were counted.

In the rotarod test, animals were trained for 2 minutes at a speed of 4 rpm. After this initial training, mice performed 8 trials of maximum 5 minutes with increasing speed starting from 4 rpm up to 40 rpm. This test was performed at 14 weeks after injection and fall off time was recorded.

In the open field test, animals were placed for 30 minutes in the dark prior to the open field cage (50×50 cm). After 1 minute of adaptation, the next 10 minutes were video-recorded for analysis. This test was performed at 15 weeks after injection.

### Histology

At selected time points, mice were deeply anesthetized using an overdose of pentobarbital (Nembutal, CEVA Santé, Belgium) and transcardially perfused with saline followed by ice-cold 4% paraformaldehyde in phosphate-buffered saline (PBS). 50-μm-thick coronal brain sections were cut with a vibrating microtome (HM650V, Microm, Germany) and stored at 4°C in 0.1% sodium azide in PBS. Immunohistochemistry was performed under uniform conditions on free-floating sections using antibodies raised against α-synuclein (rabbit polyclonal, 1:5000, Millipore 5038), tyrosine hydroxylase (TH, rabbit polyclonal, 1:5000, Millipore 152) and P-S129 α-synuclein (mouse monoclonal, 1:5000, Elan Pharmaceuticals, [31,41]). Sections were pretreated with 3% hydrogen peroxide for 10 minutes to quench endogenous peroxidase activity and then incubated overnight at room temperature in primary antibody solution with 10% normal goat serum (DakoCytomation, Belgium). As secondary antibody, we used biotinylated anti-rabbit or anti-mouse IgG (1:600, DakoCytomation), followed by incubation with streptavidin-horseradish peroxidase complex (1:1000, DakoCytomation). TH immunoreactivity was visualized using Vector SG (SK-4700, Vector Laboratories) as chromogen; and (P-S129) α-synuclein was visualized with 3,3-diaminobenzidine (0,4 mg/ml, Sigma-Aldrich). After being rinsed and mounted, the sections were cover-slipped using DPX mounting medium (Sigma).

For fluorescent double staining, sections were rinsed three times in PBS and incubated overnight in the dark in PBS-0.1% Triton X-100 and 0.1% sodium azide, 10% donkey serum and the following antibodies: chicken anti-GFP (1:500, Aves Labs 1020), rabbit anti-α-synuclein (1:1000, Millipore 5038), mouse anti-TH (1:1000, Millipore MAB318), mouse anti-Neuronal Nuclei (NeuN, 1:500, Chemicon MAB377), mouse anti-α-synuclein (1:100, Invitrogen LB309) and rabbit anti-Glial Fibrillary Acidic Protein (GFAP, 1:100, Dako Z0334). After rinsing, the sections were incubated in the dark for 1 h in fluorochrome-conjugated secondary antibodies: donkey anti-chicken FITC (1:400, Lucron), donkey anti-rabbit Alexa 647 and donkey anti-mouse Alexa 555 (1:400, Molecular Probes, Invitrogen). After being rinsed and mounted, the sections were cover-slipped with Mowiol (Sigma).

### Stereological quantification and confocal microscopy

The volume of α-synuclein expression in the SN and of TH-immunoreactive fibbers in the striatum (STR) was determined by stereological volume measurements based on the Cavalieri method as described before [42,43]. The number of TH-, α-synuclein- and P-S129 α-synuclein-positive cells in the SN was estimated using a random sampling stereological counting method, the optical fractionator [44], in a computerized system (StereoInvestigator; MicroBright-Field, Magdeburg, Germany). Coefficient of error attributable to the sampling was calculated according to Gundersen and Jensen [45]. Errors ≤0.10 were accepted. The SN was delineated from -2.54 to -3.88 mm posterior to bregma based on the Paxinos atlas for the mouse brain [40]. For each animal, every third section throughout the rostro-caudal extent of the SN and every fourth section covering the entire extent of the STR were incorporated to the counting procedure. The conditions of the experiment were blinded to the investigator.

Fluorescent double immunostainings for α-synuclein or GFP and TH, and for α-synuclein and NeuN or GFAP, were visualized by confocal laser scanning microscopy (Fluoview 1000, Olympus). Estimation of co-transduction was performed based on quantifications of double-stained cells on 4 different sections.

### Sequential protein extraction

After cervical dislocation, the SN were rapidly dissected from one to two 1 mm-thick coronal sections using a mouse brain slicer, and snap frozen in dry ice. Frozen nigral tissues were homogenized in 5 volumes of Tris-buffered saline (TBS, pH 7.5, 10 mM Tris, 0.15 M NaCl) plus complete protease inhibitor (TBS+, Roche Applied Science) using a tissue homogenizer (Ultra-Turrax; IKA Werke, Staufen, Germany). Next, proteins were sequentially extracted, slightly modified from previously published procedures [46,47]. Shortly, samples were spun at 120,000 × g for 30 minutes at 4°C. The resulting supernatant represented the TBS soluble fraction. The pellet was then resuspended in TBS+ containing 1% Triton X-100 (Sigma-Aldrich), TBS+ containing 1 M sucrose and RIPA buffer (50 mM Tris–HCl, pH 7.4, 175 mM NaCl, 5 mM ethylenediaminetetraacetic acid [EDTA], 1% NP-40, 0.5% sodium deoxycholate, and 0.1% sodium dodecyl sulfate [SDS]). After a last ultra-centrifugation step, the detergent-insoluble pellet was solubilised in 2 M urea/5% SDS to preserve α-synuclein structures [48]. Samples were supplemented with 10% glycerol and stored at -80°C.

### Western blot analysis

Protein concentration was measured using the Bradford method (Protein Assay Dye Reagent Concentrate, Biorad, Munich, Germany). For western blotting, 30 μg of fractionated protein extracts were loaded on 12% acrylamide gel (Serva, Heidelberg, Germany) and blotted onto a methanol-activated PVDF membrane (Immobilon P, Millipore Corporation, Billerica, MA, USA). Immunoblots were blocked in 5% dry milk in TBST buffer (TBS plus 0.1% Tween 20) and subsequently probed with: human specific anti-α-synuclein 15G7 (1:10, AG Scientific), anti-α-synuclein Syn1 detecting both mouse and human α-synuclein (1:1000, BD Bioscience), anti-P-S129 α-synuclein (1:1000, Epitomics, 2014–1). Anti-GAPDH (1:1000, Abcam 125247) or anti-β-actin (1:10000, Sigma A5441) was used as internal loading control. Bound antibodies were detected with horseradish peroxidase-conjugated secondary antibodies and enhanced chemiluminescence (ECL or ECLplus, GE Healthcare) followed by exposure to hyperfilm (GE Healthcare). ImageJ software was used to determine the optical density of protein bands and all data were analyzed for each viral vector group (n = 3) based on 3 independent blots and normalized to the expression of the corresponding GAPDH or β-actin loading control within line.

### Statistical analysis

Statistical analysis was performed using the GraphPad Prism 5.0 software package. Results are expressed as means ± standard error of the mean. Statistical significance was assessed using two-way ANOVA followed by Bonferroni post-hoc test for intergroup comparisons or one-way ANOVA for the behavioural tests and the western blotting analysis. Statistical significance level was set as follows: * if P < 0.05, ** if P < 0.01, *** if P < 0.001.

## Results

### Efficient transduction of dopaminergic neurons upon rAAV2/7 vector-mediated overexpression of α-synuclein or eGFP in mouse SN

**Figure 1:**
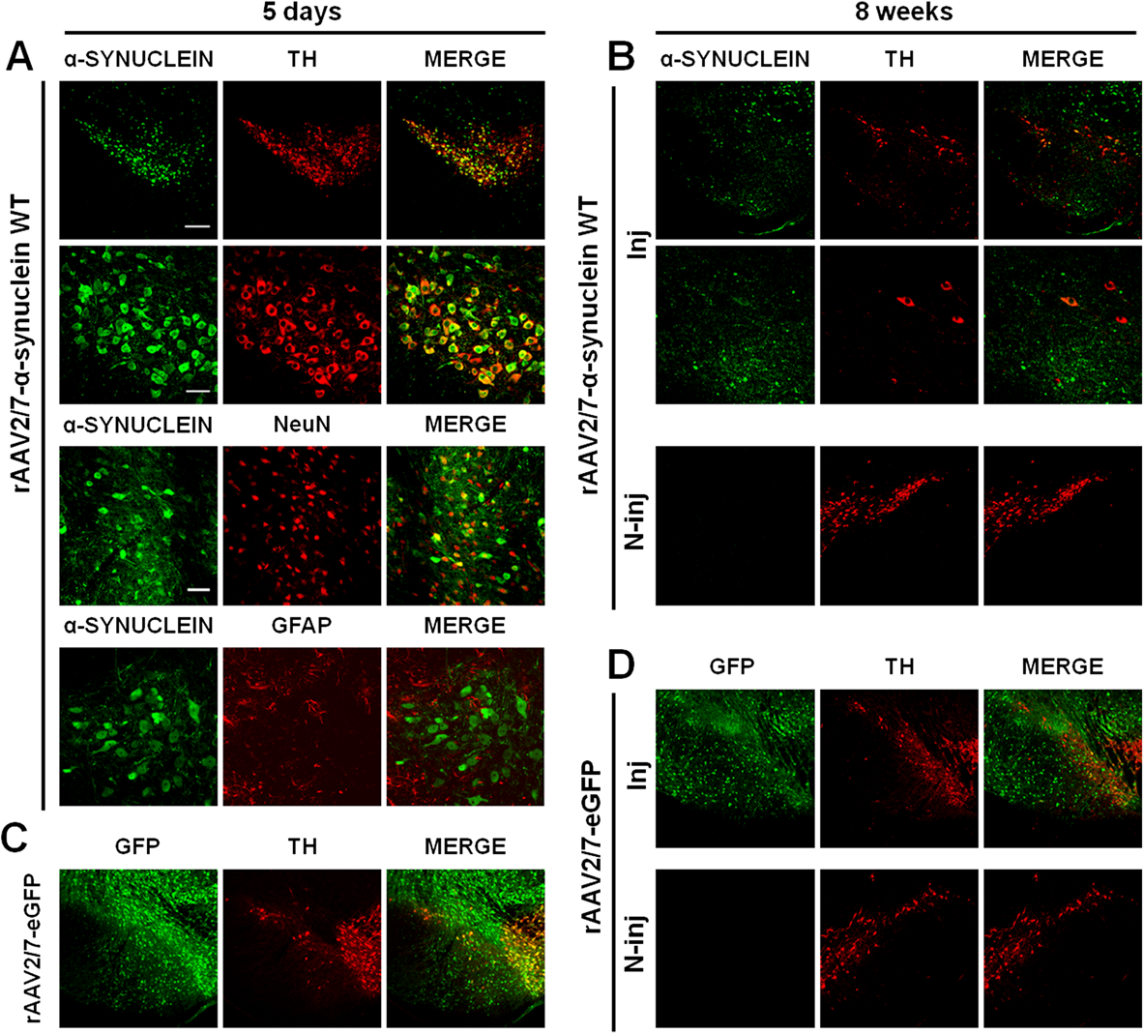
**Efficient dopaminergic neuron transduction upon rAAV2/7 vector delivery in mouse SN. (A-B)** Representative confocal images of fluorescent double immunostainings for α-synuclein (green) and TH (red) at **(A)** 5 days and at **(B)** 8 weeks after injection of rAAV2/7-α-synuclein WT at 8,0E + 11 GC/ml. **(A)** At 5 days post-injection, pictures reveal extensive co-localization (merge) in the transduced region. Bottom panels are magnifications of the overviews (upper panels). Scale bar upper panel = 200 μm and bottom panel = 50 μm. Confocal images of double immunostainings for α-synuclein (green) and NeuN or GFAP (red) show an almost exclusive neuronal transduction. Scale bar = 100 μm. **(B)** At 8 weeks after injection, a clear degeneration of the nigral dopaminergic neurons is observed upon rAAV2/7 vector-mediated α-synuclein overexpression. Dopaminergic neurons remain widely present in the contralateral side. **(C-D)** Fluorescent double stainings for GFP (green) and TH (red) at **(C)** 5 days and at **(D)** 8 weeks post-injection show that rAAV2/7-eGFP at 8,0E + 11 GC/ml does not induce any dopaminergic cell death over time. Inj: injected side; N-inj: non-injected side.

rAAV2/7-α-synuclein WT and rAAV2/7-eGFP vectors were stereotactically injected in mouse SN (2 μl of 8,0E + 11 GC/ml). We performed fluorescent double immunostainings to assess transgene expression (Figure 1). At 5 days post-injection, widespread transgene expression was evident in the injected side for both α-synuclein and eGFP viral vectors (Figure 1A, C). Transgene expression of eGFP appeared stronger than α-synuclein expression at comparable vector doses, although this might be due to differential sensitivities of the respective antibodies. Co-localization of α-synuclein or GFP with the dopaminergic marker tyrosine hydroxylase (TH) indicated efficient transduction of the nigral dopaminergic neurons distributed throughout the SN pars compacta. Approximately 85% of the dopaminergic neurons in SN were transduced by the viral vectors as assessed by high magnification confocal imaging (Figure 1A). In addition, double immunostaining for α-synuclein and the neuron-specific nuclear marker NeuN clearly indicated efficient transduction of neurons in the injected area, around 95% of double-positive cells, whereas double immunostainings for α-synuclein and the astrocytic marker GFAP showed an absence of co-localization, pointing to an almost exclusive neuronal transduction.

In order to evaluate the sustained nigral viral vector-mediated transgene expression, we performed fluorescent double immunostainings at 8 weeks post-injection (Figure 1B, D). For rAAV2/7-eGFP, we observed a stable transgene expression and normal TH immunoreactivity in the SN (Figure 1D). In contrast, we detected few surviving α-synuclein- and TH-positive cells for rAAV2/7-α-synuclein WT, indicative of a significant loss of both α-synuclein- and TH-positive cells at this time point in mouse SN (Figure 1B). The non-injected sides showed no transgene expression (α-synuclein or GFP) or loss of dopaminergic neurons (TH) at any time point after injection of rAAV2/7-α-synuclein WT or rAAV2/7-eGFP.

### rAAV2/7 vector-mediated overexpression of α-synuclein in mouse SN causes dose-dependent progressive cell death and α-synuclein aggregation

**Figure 2:**
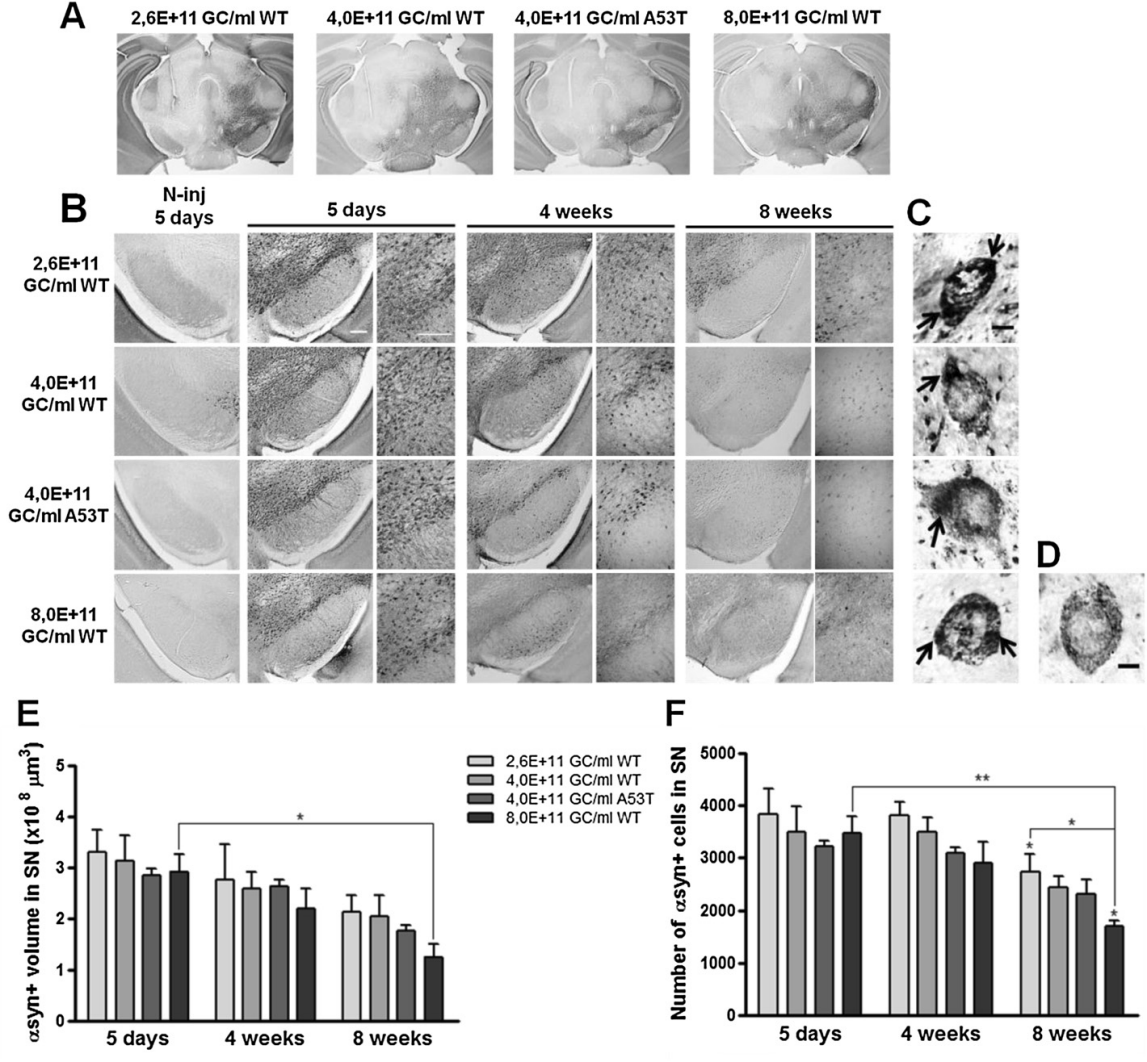
**rAAV2/7 vector-mediated overexpression of α-synuclein in mouse SN induces progressive cell loss and α-synuclein aggregation. (A)** Images of immunohistochemical stainings for α-synuclein showing the whole area of transduction 5 days after rAAV2/7-α-synuclein injection. Scale bar = 500 μm. **(B)** Overexpression of WT α-synuclein induces a progressive and dose-dependent α-synuclein-positive cell loss over time in the injected SN. Absence of immunoreactivity in the contralateral SN at 5 days after injection when expression was maximal. Right panels are magnifications of the overview (left panels). Scale bars = 200 μm. N-inj: non-injected side. **(C)** High magnification pictures demonstrate the presence of α-synuclein-positive aggregates (arrows) in the SN for each vector dose at 4 weeks post-injection. **(D)** High magnification picture of an α-synuclein-positive cell without aggregates. Scale bars **(C-D)** = 5 μm. **(E)** Stereological quantification of the α-synuclein-positive volume in the SN of mice injected with 3 different WT α-synuclein vector doses and a unique A53T α-synuclein vector dose after 5 days, 4 weeks and 8 weeks. **(F)** Stereological quantification of the number of α-synuclein-positive cells in the SN of mice after 5 days, 4 weeks and 8 weeks. Asterisks (*) depict significant decrease respective to 4 weeks, unless specified otherwise. 5 days 4,0E + 11 GC/ml WT/A53T: n = 3; 5 days 2,6/8,0E + 11 GC/ml WT: n = 4; 4 weeks: n = 4; 8 weeks: n = 4.

In order to evaluate the dose- and time-dependent effects of α-synuclein overexpression, rAAV2/7-α-synuclein WT vector injections were unilaterally performed in adult mouse SN at 3 different vector titers: 2,6E + 11 GC/ml; 4,0E + 11 GC/ml and 8,0E + 11 GC/ml. Mice were perfused for immunohistochemical analysis at 5 days, 4 weeks and 8 weeks after surgery. Staining of the nigral sections for human α-synuclein revealed widespread expression of the transgene in the injected area after 5 days (Figure 2A-B). In addition, we observed a progressive reduction in α-synuclein expression over time in the injected region for all vector doses tested, and the loss was faster and stronger with increasing viral vector titer (Figure 2B).

A cardinal feature in the neuropathology of PD is the presence of cytoplasmic α-synuclein-rich inclusions in the remaining surviving neurons. Therefore, we investigated the presence of α-synuclein-positive cells exhibiting LB-like aggregates in the cytoplasm. α-Synuclein-positive cells with aggregates were visualized with high magnification resolution imaging and selected on the presence of dark inclusions of α-synuclein protein within the cytoplasm and an overall dystrophic morphology, as opposed to cells without aggregates (Figure 2C-D). At 4 weeks post-injection, we detected α-synuclein-positive cells with aggregates for each vector dose tested.

Next, we quantified the α-synuclein expression in the injected SN at the different time points by means of two stereological methods (Figure 2E-F). First, we determined the α-synuclein-positive transduced volume in the SN (Figure 2E). We observed a progressive decrease over time for all viral vector doses. At 8 weeks post-injection, this reduction in the nigral α-synuclein-positive volume was clearly more pronounced when the viral vector dose increased. Subsequently, we quantified the number of individual α-synuclein-positive cells in the SN and we observed a similar gradual and dose-dependent decrease in α-synuclein expression over time as observed for the α-synuclein-positive transduced volume (Figure 2F).

In addition, we aimed at comparing the overexpression of mutant A53T α-synuclein in mouse SN with WT α-synuclein. For this approach, we injected rAAV2/7-α-synuclein A53T in mouse SN at the same intermediate viral vector titer of 4,0E + 11 GC/ml. Both viral vectors were produced in parallel, normalized for the same GC/ml and delivered in the same manner. We observed that rAAV2/7-α-synuclein A53T also triggered a progressive reduction in α-synuclein expression and the formation of cytoplasmic α-synuclein-positive aggregates over time (Figure 2B-C). Although not significant, the extent of neurodegeneration was slightly stronger than for the WT α-synuclein viral vector (Figure 2E-F).

### rAAV2/7 vector-mediated overexpression of α-synuclein induces progressive nigral and striatal dopaminergic neurodegeneration in a dose-dependent manner

**Figure 3:**
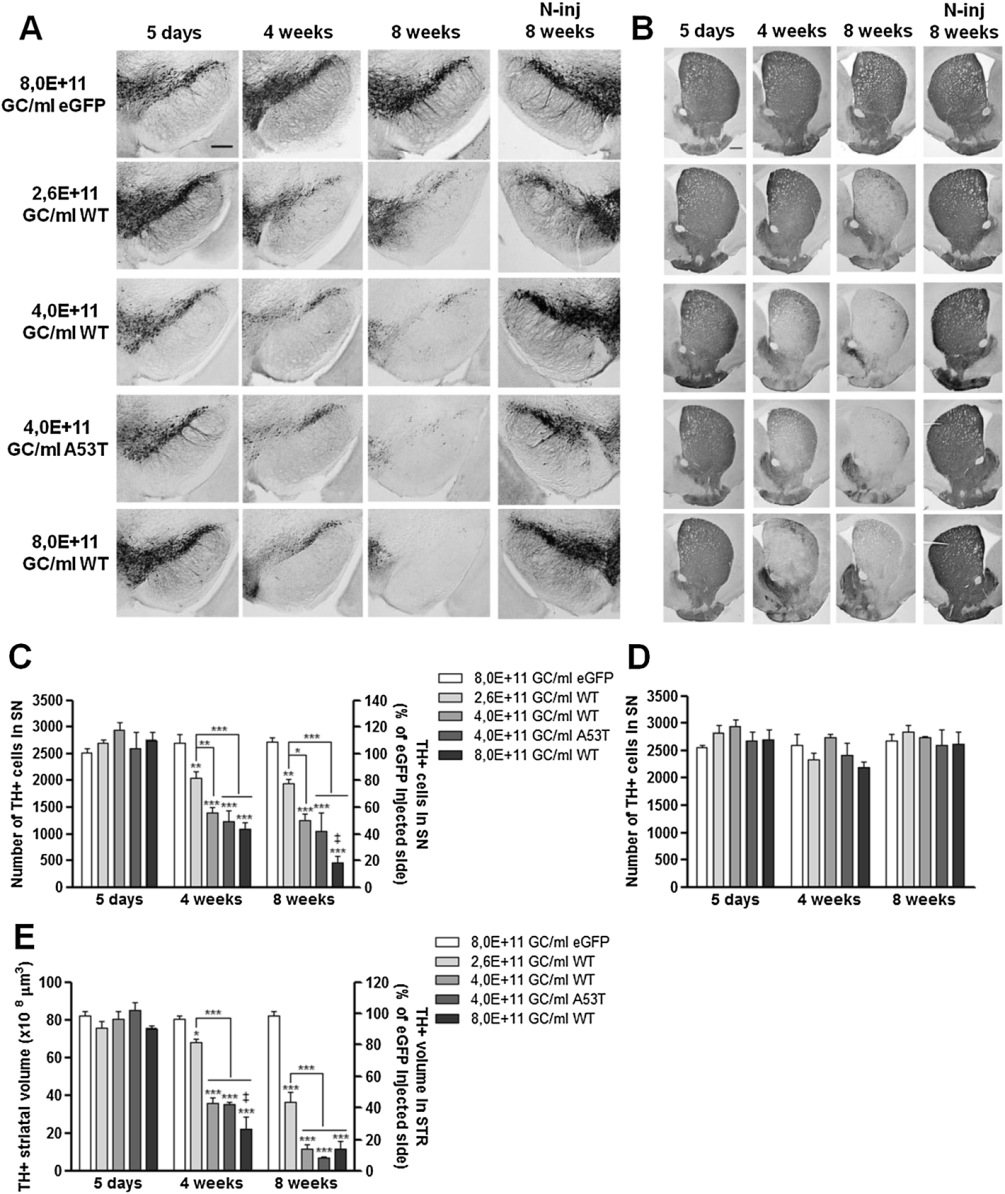
**α-Synuclein overexpression leads to progressive dose-dependent dopaminergic cell death in mouse SN and STR. (A-B)** Immunohistochemical stainings for TH in the **(A)** SN and in the **(B)** STR of mice injected with 3 different WT α-synuclein vector doses and a unique eGFP or A53T α-synuclein vector dose show that overexpression of α-synuclein induces a progressive and dose-dependent TH cell loss over time. Overexpression of A53T α-synuclein triggers a comparable loss to WT α-synuclein at the same vector dose. Note the absence of dopaminergic neurodegeneration in the contralateral side at 8 weeks post-injection when cell loss was maximal. Scale bar **(A)** = 250 μm and **(B)** = 500 μm. N-inj: non-injected side. **(C-D)** Stereological quantification of the number of TH-positive neurons in the SN of the **(C)** injected side and **(D)** non-injected side after 5 days, 4 weeks and 8 weeks. **(E)** Stereological quantification of the TH-positive volume in the STR of the injected side at 5 days, 4 weeks and 8 weeks post-injection. Asterisks (*) represent significant decrease respective to eGFP, unless specified otherwise. Dagger (‡) depicts significant decrease respective to 4,0E + 11 GC/ml WT/A53T. 5 days 8,0E + 11 GC/ml eGFP and 4,0E + 11 GC/ml WT/A53T: n = 3; 5 days 2,6/8,0E + 11 GC/ml WT: n = 4; 4 weeks: n = 4; 8 weeks: n = 4.

To specifically evaluate the dopaminergic neurodegeneration induced by the overexpression of α-synuclein at the different viral vector doses, we performed immunohistochemical stainings for TH in the SN. As control, the rAAV2/7-eGFP vector was tested at the highest vector dose used in this study (8,0E + 11 GC/ml) to exclude unspecific toxic effects due to the high viral vector titer. We observed a progressive and significant loss of nigral dopaminergic neurons over time for all doses of α-synuclein vector (Figure 3A). In contrast, the rAAV2/7-eGFP vector did not induce any nigral dopaminergic neurodegeneration in this time frame. In addition, no dopaminergic cell loss was observed at the contralateral side at 8 weeks post-injection when the neurodegeneration was maximal. Next, we estimated the total number of TH-positive neurons in the SN by means of unbiased stereological quantification (Figure 3C). At 5 days post-injection, no acute cell death was observed for any of the different viral vectors. As for the lowest α-synuclein viral vector dose tested (2,6E + 11 GC/ml), stereological counts revealed a 19% TH-positive neuron loss at 4 weeks after injection respective to rAAV2/7-eGFP, which increased up to 23% at 8 weeks post-injection. Nigral dopaminergic neurodegeneration was also observed for the other two WT α-synuclein viral vector titers: from 45% at 4 weeks to 50% at 8 weeks for the intermediate vector dose (4,0E + 11 GC/ml); and from 57% at 4 weeks to 82% at 8 weeks for the highest dose (8,0E + 11 GC/ml). Stereological quantification of the number of TH-positive cells in the SN contralateral to the side of injection showed no cell death for all conditions (Figure 3D). These results show that a progressive dopaminergic cell death is observed over time for all α-synuclein vector conditions, and that the degree of dopaminergic neuron loss at a given time point positively correlates with the viral vector dose used.

Similar to the observations for the SN, immunohistochemical stainings for TH in the STR revealed a gradual decrease in TH expression over time for the 3 different α-synuclein WT vector doses whereas the non-injected side remained intact (Figure 3B). Next, we assessed the degeneration of the dopaminergic nerve terminals in the STR by stereological quantification of the striatal TH-positive volume. At 4 weeks post-injection, stereological counts revealed a 17% TH-positive volume reduction respective to the contralateral side for the lowest vector dose (2,6E + 11 GC/ml), that increased up to 56% at 8 weeks post-injection (Figure 3D). The same progressive loss over time was observed for the intermediate vector dose (4,0E + 11 GC/ml): from 56% at 4 weeks to 86% at 8 weeks; and for the highest vector dose (8,0E + 11 GC/ml): from 73% at 4 weeks to 86% at 8 weeks. In contrast, delivery of the rAAV2/7-eGFP in the SN of mice did not cause any neurodegeneration in the STR (Figure 3B, E). Here again, the neurodegenerative effect became stronger when the viral titer increased.

We also compared WT and mutant A53T α-synuclein in their potency to induce dopaminergic neuron loss over time. Similar to the trends observed in Figure 2, the nigral dopaminergic cell death caused by the delivery of A53T α-synuclein was progressive over time and slightly enhanced, although not significantly different, when compared to the WT α-synuclein vector at the same vector dose. The dopaminergic cell loss in the SN varied from 51% at 4 weeks after injection to 59% at 8 weeks (compared to 45% at 4 weeks and 50% at 8 weeks for WT α-synuclein). As for the STR, 57% of the TH-positive volume was lost at 4 weeks post-injection and 91% at 8 weeks (compared to 56% at 4 weeks and 86% at 8 weeks for WT α-synuclein).

### rAAV2/7 vector-mediated overexpression of α-synuclein in mouse SN induces progressive phosphorylation of α-synuclein at serine 129

**Figure 4:**
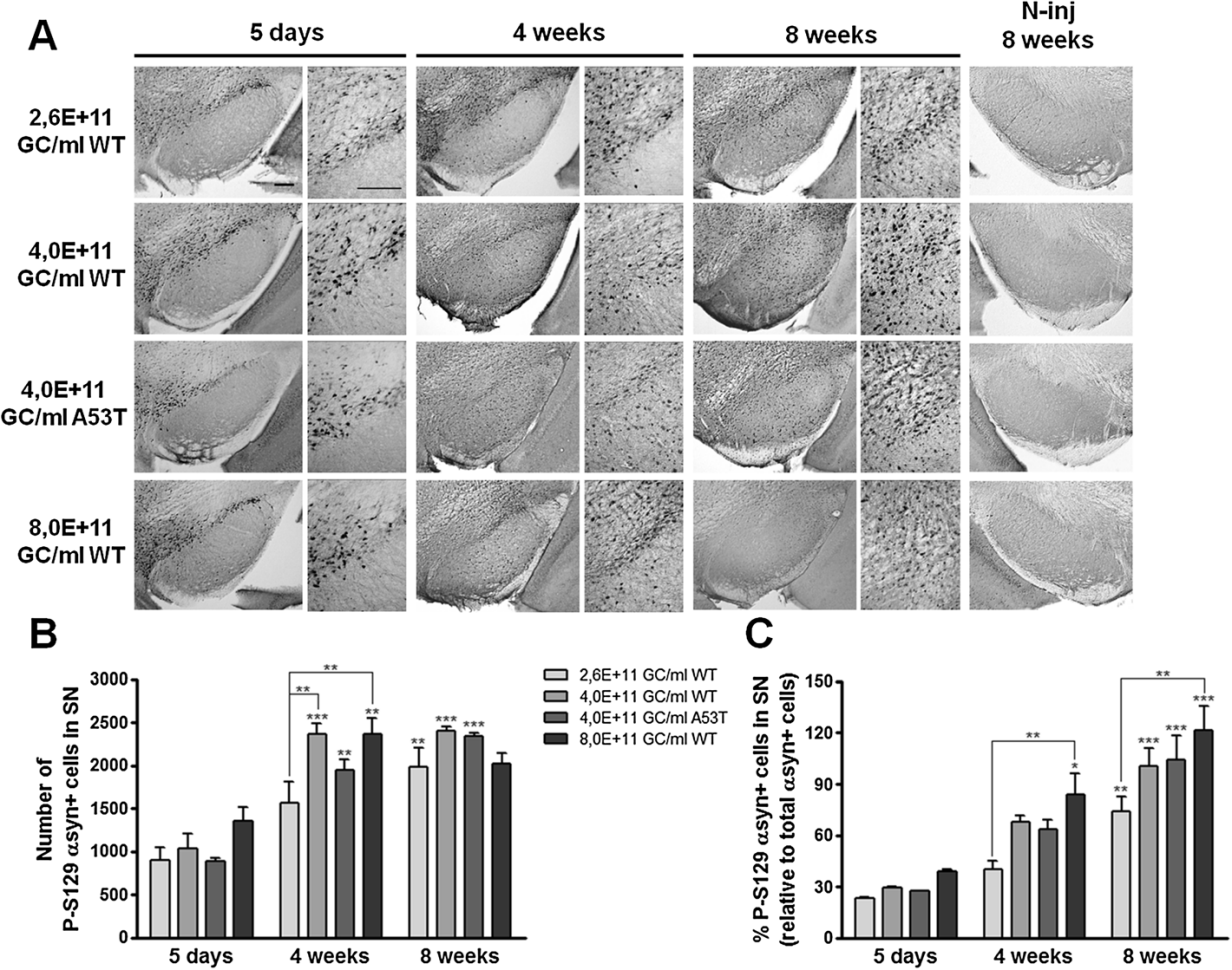
**Phosphorylation of α-synuclein at S129 increases over time in a dose-dependent manner after rAAV2/7-α-synuclein delivery. (A)** Representative images of P-S129 α-synuclein expression in the SN of mice injected with 3 different WT α-synuclein vector doses and a unique A53T α-synuclein vector dose show that overexpression of WT α-synuclein induces a progressive and dose-dependent increase over time in P-S129 α-synuclein. Lack of immunoreactivity in the contralateral SN at 8 weeks after injection when expression was maximal. Right panels are magnifications of the overview (left panels). Scale bars = 200 μm. **(B)** Stereological quantification of the number of P-S129 α-synuclein positive cells in the injected SN after 5 days, 4 weeks and 8 weeks. **(C)** Percentage of P-S129 α-synuclein positive cells in the SN at 5 days, 4 weeks and 8 weeks post-injection. Asterisks (*) depict significant increase respective to 5 days, unless specified otherwise. 5 days 4,0E + 11 GC/ml WT/A53T: n = 3; 5 days 2,6/8,0E + 11 GC/ml WT: n = 4; 4 weeks: n = 4; 8 weeks: n = 4.

Previous studies have shown that approximately 90% of the α-synuclein accumulated in the LBs is phosphorylated at serine residue at position 129 (P-S129) [30,31]. Therefore, it is considered a marker of α-synuclein neuropathology [49]. In order to further characterize the neuropathology induced by the overexpression of α-synuclein in mouse SN, we performed immunohistochemical stainings for P-S129 α-synuclein (Figure 4A). Overall, we observed a net increase over time in the number of P-S129 α-synuclein-positive cells after injection of either rAAV2/7-α-synuclein WT or A53T viral vectors while the contralateral side showed no apparent immunoreactivity. A maximum of P-S129 α-synuclein-positive cells was reached at 4 weeks post-injection by WT α-synuclein vector delivered at 4,0E + 11 or 8,0E + 11 GC/ml (Figure 4B). At 8 weeks after injection, the number of positive cells at the intermediate dose remained invariable, while a trend to a decrease was observed for the highest dose, suggesting that the vast cell loss observed at this time point affects the P-S129 α-synuclein counts. For the lowest α-synuclein vector dose (2,6E + 11 GC/ml), a more moderate and delayed increase in the number of nigral P-S129 α-synuclein-positive cells was observed over time, in accordance with the lower rate of neurodegeneration achieved by this dose. Interestingly, the A53T α-synuclein condition exhibited a somewhat lower number of nigral P-S129 α-synuclein-positive cells at 5 days and 4 weeks when compared to the WT α-synuclein viral vector.

We also estimated the percentage of P-S129 α-synuclein-positive cells in the SN by determining the ratio between the number of P-S129 α-synuclein-positive cells (Figure 4B) and the total number of α-synuclein-positive cells (Figure 2F) in the SN. For all vector doses, we observed a progressive increase over time in the percentage of P-S129 α-synuclein-positive cells in the SN (Figure 4C). Once more, we noted a dose-dependency effect given that the increase in the percentage of P-S129 α-synuclein-positive cells was significantly different from low to high vector dose at both 4 and 8 weeks after injection.

### rAAV2/7 vector-mediated overexpression of WT or A53T α-synuclein induces a progressive expression and accumulation of human α-synuclein

To examine in more detail the nature and solubility of the different α-synuclein species induced by viral vector delivery, rAAV2/7 vectors encoding WT or A53T α-synuclein, or eGFP as control, were injected in the mouse SN at the unique vector titer of 4,0E + 11 GC/ml. To separate most soluble from insoluble proteins, nigral tissues were sequentially extracted using buffers with different salt and detergent concentration, followed by intermittent ultracentrifugation steps [46].

**Figure 5:**
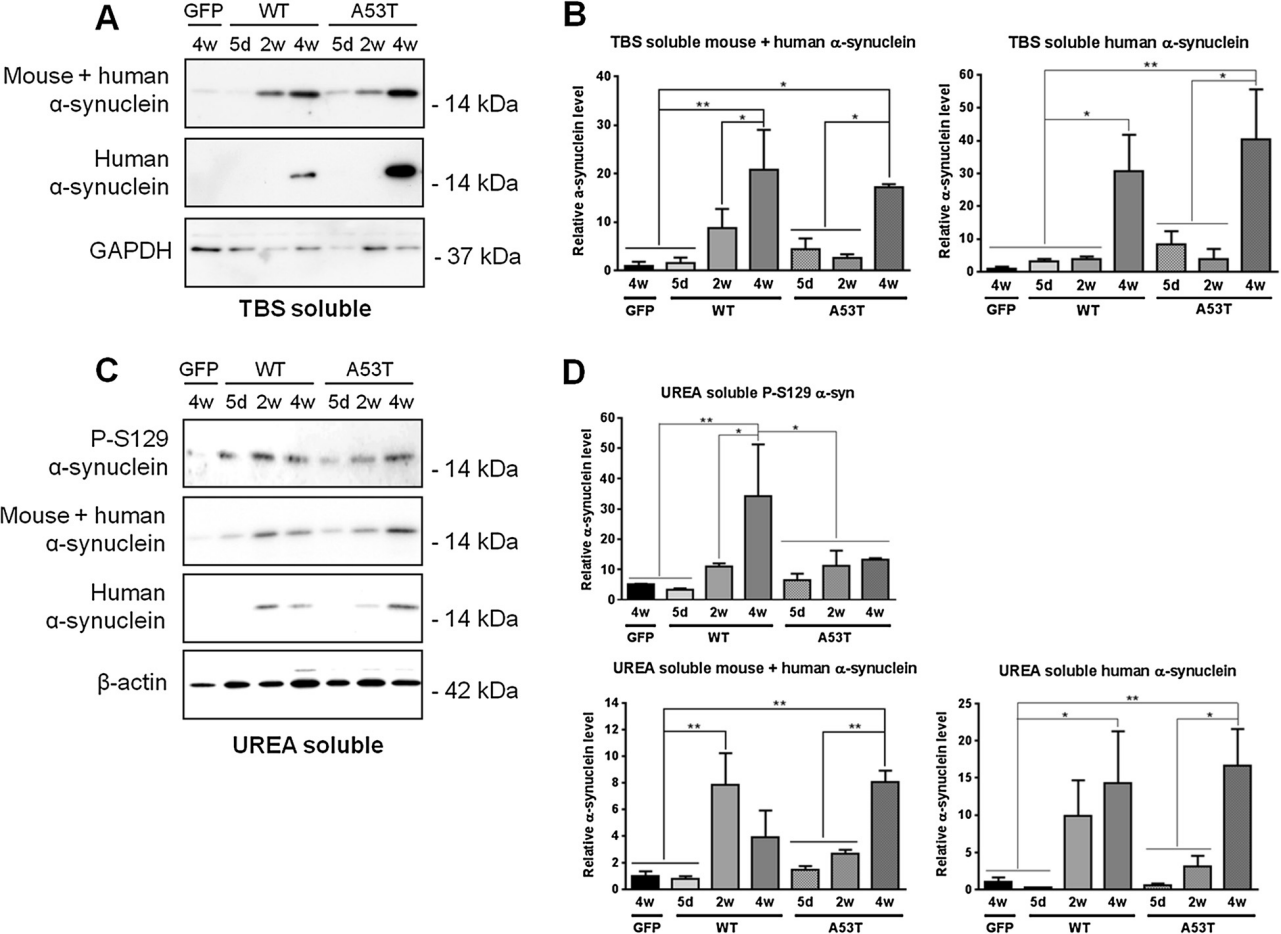
**Soluble and insoluble α-synuclein levels increase over time upon delivery of rAAV2/7-α-synuclein WT or A53T. (A)** Western blot and **(B)** quantitative analysis (n = 3) of soluble α-synuclein in the cytoplasmic (TBS soluble) fraction of SN mouse lysates injected with rAAV2/7-eGFP (4 weeks time point) or rAAV2/7-α-synuclein WT or A53T (5 days, 2 weeks and 4 weeks time points). **(C)** Immunoblotting and **(D)** quantification (n = 3) of phosphorylated and detergent-insoluble α-synuclein in the Urea soluble fraction of injected mouse nigral homogenates. For detection, immunoblots for α-synuclein were performed using a panel of different antibodies against: mouse and human α-synuclein (Syn1), specific human α-synuclein (15G7) and P-S129 α-synuclein. GAPDH and β-actin serve as internal loading control in the TBS and Urea fractions, respectively.

Samples were analyzed on Western blot in order to identify changes in the solubility of human α-synuclein encoded by the rAAV2/7-α-synuclein vector. We detected an up-regulation of α-synuclein using both an antibody recognizing mouse and human α-synuclein (Syn1, aa 91–96) and a human-specific antibody (15G7, aa 116–131). We observed a significant increase in soluble human α-synuclein at 4 weeks after injection (~14 kDa) in the cytoplasmic (TBS soluble) fraction for both WT and A53T viral vectors (Figure 5A-B). Coherently, detergent-insoluble α-synuclein detected in the Urea soluble fraction was also markedly increased over time for both WT and A53T viral vectors (Figure 5C-D). In the two isolated fractions, human α-synuclein was undetectable in all immunoblots of the rAAV2/7-eGFP control vector at 4 weeks time point (Figure 5A, C).

Additionally, in the Urea soluble fraction, we observed an increase in P-S129 α-synuclein between 5 days and 2 weeks for both α-synuclein vectors, although the increase was more pronounced in the WT condition, which is in agreement with our immunohistochemical data (Figure 5C-D).

### Motor behaviour impairments induced by rAAV2/7 vector-mediated overexpression of α-synuclein in mouse SN

**Figure 6:**
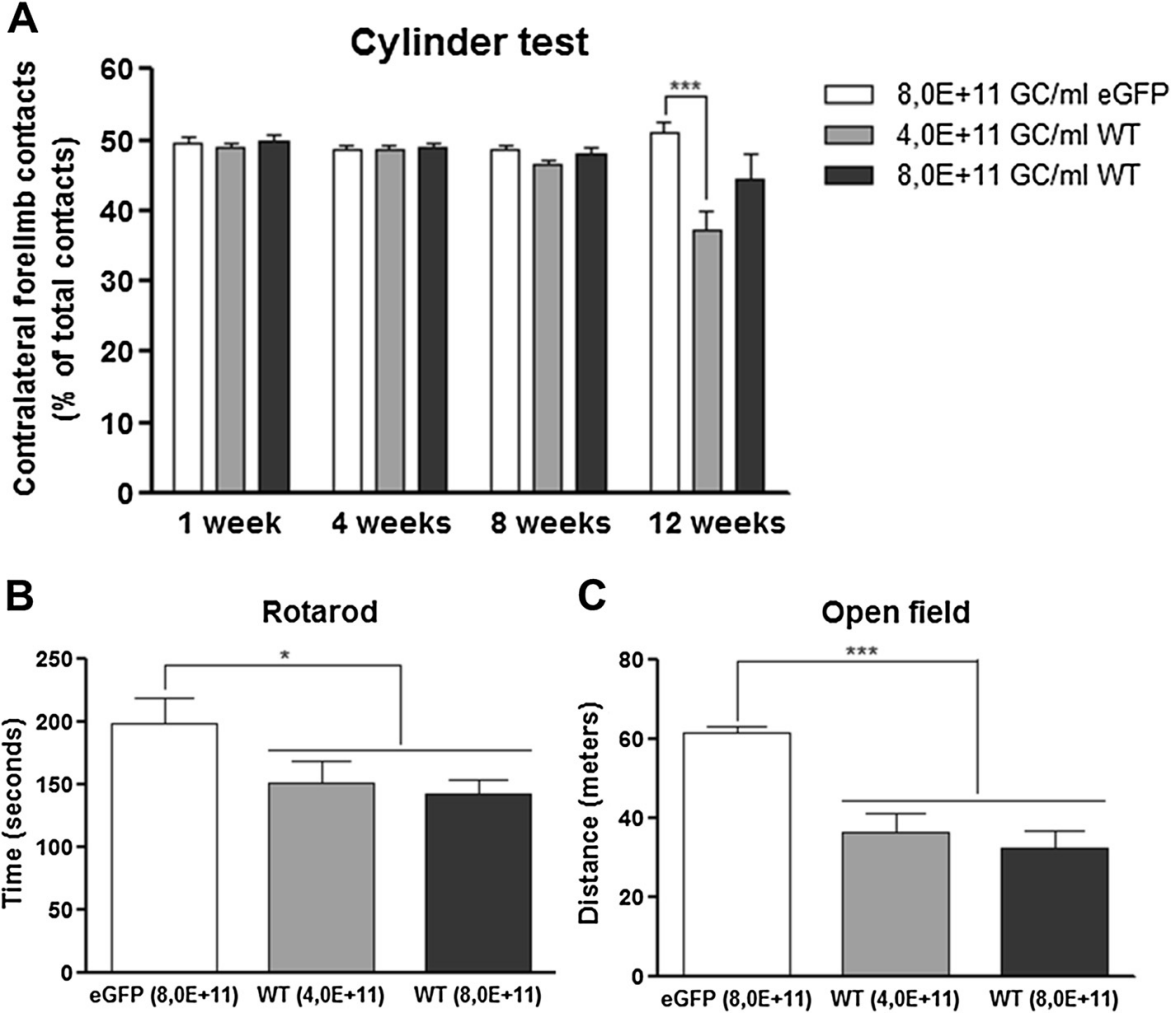
**rAAV2/7 vector-mediated α-synuclein overexpression in mouse SN leads to motor behaviour impairments. (A)** Performance in the cylinder test of rAAV2/7-eGFP and rAAV2/7-α-synuclein WT mice at 1 week, 4 weeks, 8 weeks and 12 weeks after injection. No asymmetry in forepaw use was detected up to 8 weeks. At 12 weeks after injection, a significantly reduced use of the contralateral forepaw was observed for the mid dose of α-synuclein vector. **(B)** The rotarod test performed at 14 weeks after injection showed a significant reduction in average time on the rotating rod for both rAAV2/7-α-synuclein doses. **(C)** A significant decline in the walking distance was observed for the two α-synuclein doses tested in the open field at 15 weeks after injection. For all tests: 8,0E + 11 GC/ml eGFP/WT and 4,0E + 11 GC/ml WT: n = 10.

Finally, we wondered whether the progressive dopaminergic cell death triggered by the overexpression of α-synuclein in the SN would affect the motor behaviour of the mice. We first evaluated the performance of the mice in the cylinder test. Somewhat surprisingly, no significant differences between left and right forelimb use were detected up to 8 weeks after injection (Figure 6A). Therefore we decided to retest the animals at a later time point. At 12 weeks after injection, a small but significant decrease in the use of the forelimb contralateral to the lesion was observed with the medium dose of α-synuclein (Figure 6A). However, since disturbances in behaviour were clearly visible from the video-recordings, we used two other motor tests. In the rotarod test executed at 14 weeks after injection, both α-synuclein groups, mid and high dose, performed significantly worse than the eGFP control group (Figure 6B). Similarly, in the open field test carried out at 15 weeks after injection, both rAAV2/7-α-synuclein injected groups of mice walked over a considerably reduced distance compared to the eGFP control group (Figure 6C).

## Discussion

The goal of the current study was to develop a robust mouse model that displays the most relevant neuropathological hallmarks of PD based on the targeted viral vector delivery of α-synuclein to the SN. We showed that rAAV2/7 vector-mediated overexpression of either WT or mutant A53T α-synuclein results in an overt degeneration of the nigral dopaminergic neurons concomitant with the development of motor impairments and the formation of cytoplasmic α-synuclein inclusion bodies in a particularly short time frame.

The identification of α-synuclein as a key factor in idiopathic and inherited PD has impelled the development of new mouse models overexpressing α-synuclein over the last decade. However, transgenic mouse lines have failed to reproduce convincingly a meaningful progressive dopaminergic neurodegeneration in the SN [12]. In contrast, local delivery of viral vectors overexpressing α-synuclein in rat and non-human primate brains leads to gradual nigral dopaminergic neuron loss, which is in general more prominent with rAAV vectors compared to lentiviral vectors [50]. However, the rAAV vector-based mouse models published to date exhibit no or limited dopaminergic cell loss with a maximum of 20 to 35% between 2 and 6 months post-injection [33-38]. Moreover, these mouse models do not display striatal dopaminergic fiber loss [35,36]. We have previously reported that lentiviral vector-mediated overexpression of WT or mutant A30P α-synuclein in mouse SN induces up to 25% dopaminergic neurodegeneration at 10 to 12 months after injection [16]. The long time scope needed to achieve a substantial neurodegeneration differs from our new mouse model developed in the present study in the type of viral vector used. rAAV vectors are more efficient than lentiviral vectors in transducing the dopaminergic neurons of the SN because of their high titers and neuronal tropism. In this regard, it is remarkable that a very recent study using lentiviral vectors for the overexpression of α-synuclein in mouse SN has reported around 60% dopaminergic cell loss after 45 days for the A30P, A53T and E46K mutant and about 25% for WT α-synuclein [51].

The experience with viral vector-based rat models has proved that transduction efficiency and transgene expression levels greatly determine the degree of nigral neuron degeneration and the time span over which this occurs [52,53]. Consequently, we have previously optimized large-scale and flexible manufacturing processes for rAAV vector production [54]. In addition, we have shown that our improved rAAV vectors efficiently transduce nigral dopaminergic neurons [19]. Based on this comparative study, we selected the rAAV2/7 serotype for overexpression of α-synuclein because it combines high transgene expression levels and high transduction efficiency of the dopaminergic neurons *in vivo*. We optimized a rat model of PD based on rAAV2/7 vector-mediated overexpression of A53T α-synuclein in the SN. Rats receiving a vector dose of 3,0E + 11 GC/ml displayed 80% loss of the nigral dopaminergic neurons at 4 weeks post-injection (Van der Perren et al., submitted). In parallel to that study, we have shown here that targeted nigral overexpression of WT α-synuclein by means of a rAAV2/7 vector induces up to 82% dopaminergic cell death in mouse SN within 2 months. The extent of cell loss for the whole population of α-synuclein overexpressing cells (maximum of about 50% at 8 weeks) is lower than the loss of TH-positive neurons (82% at 8 weeks with the highest titer), which suggests that the dopaminergic neurons are more vulnerable to α-synuclein neurotoxicity.

α-Synuclein pathology is the second major neuropathological feature of PD after the progressive dopaminergic cell loss. Therefore, it is an essential characteristic to replicate in a relevant animal model of PD. However, the rAAV vector-based mouse models for α-synuclein reported in literature until now do not present detectable α-synuclein-positive aggregates as far as 6 months post-injection [35]. Other studies with rAAV overexpression of α-synuclein in rats and non-human primates demonstrate the presence of α-synuclein-rich inclusions [23-25,28,29,55]. Both our new rAAV2/7-based and previous lentiviral vector-based mouse models for α-synuclein exhibited visible inclusions abundant in α-synuclein protein in the surviving neurons [16,17]. In addition, we here demonstrated increasing levels of insoluble transgenic α-synuclein over time. We also observed a gradual increase in the percentage of P-S129 α-synuclein-positive cells in the SN, supporting the progressive development of α-synuclein neuropathology and the presence of LB-like α-synuclein-rich inclusions.

Another objective of the current study was to test a dose dependency of viral vector-mediated overexpression of α-synuclein. For this approach, we used 3 different doses of WT α-synuclein vector in mouse SN that led to a dose-dependent and gradual neurodegeneration over time. With our lowest vector dose, we achieved a similar degree of dopaminergic cell death as previous studies in mice [33,35,37], while the highest dose tested induced extensive (57%) nigral dopaminergic neurodegeneration in just 4 weeks. To our knowledge, this is the first dose dependence study of viral vector overexpression of α-synuclein carried out in mouse brain. Similar comparative studies have been performed in rats, which also reported differences in rates of neurodegeneration in the SN and in behavioural performance based on the viral vector titer used [28,52,53,56].

In the present study, we investigated the behavioural performance of the mice at two different doses of WT α-synuclein viral vector. Somewhat surprisingly, the cylinder test showed only a minor asymmetric forepaw use at 12 weeks after injection compared to the eGFP control group, while all previous time points did not reveal any impairment. In an independent study, we also did not observe impairments in spontaneous forelimb use in mice with ~50% TH cell loss achieved at 4 weeks post-injection by overexpression of rAAV2/7-α-synuclein WT in mouse SN (Oliveras-Salvá et al., submitted). These observations are in contrast with own previous results obtained in rats where the same level of dopaminergic neuron loss resulted in a clear decrease in contralateral forepaw use (Van der Perren et al., submitted). However, careful observation of the mice in the cylinder test revealed a general behavioural impairment and slowness of movement. Therefore, we also performed the rotarod and open field tests which showed pronounced motor behaviour deficits in both α-synuclein groups at a later time point. These impairments can be ascribed to the extensive dopaminergic cell death obtained in mouse SN after the injection of the rAAV2/7-α-synuclein vector.

Finally, we evaluated whether the overexpression of the A53T clinical mutant triggered a substantially different degeneration of the dopaminergic cells in the SN compared to the WT α-synuclein. Overall, the neurodegeneration induced over time by the A53T α-synuclein viral vector in the SN and the STR was slightly enhanced when compared to the WT α-synuclein vector, although the difference was never significant. To date, there is no consensus in literature about the neurotoxicity of mutant A53T α-synuclein in comparison to WT α-synuclein *in vivo*. This is partly due to the scarcity of parallel comparative studies in which WT and A53T α-synuclein are investigated under the same conditions. Other studies have compared human WT and mutant A53T α-synuclein encoded by viral vectors in other species. On the one hand, Eslamboli and colleagues reported that the A53T variant induces a significantly enhanced dopaminergic neuron loss when compared to WT at one year after rAAV nigral injection in non-human primate brain and, correlating with the degree of induced cell death, two different microglia activation profiles were reported [55,57]. On the other hand, other studies performed in rats and non-human primates showed that rAAV vector-based delivery of WT and A53T α-synuclein induces a comparable neurodegenerative process, as well as aggregate formation [23,29,56]. Our findings in the present study are in agreement with these latter publications. In mice, we have previously reported that overexpression of WT and A30P α-synuclein by means of lentiviral vectors induces a similar rate in dopaminergic cell death and comparable α-synuclein pathology in a long-term study of 12 months [16]. In our hands, the overexpression levels rather than the clinical mutation determine the neuropathological changes in our model.

## Conclusions

To conclude, we have developed a robust α-synuclein mouse model by means of rAAV2/7-vector delivery which recapitulates several indispensable features of a PD animal model: motor deficits, robust and progressive nigral dopaminergic neuron loss over time and the presence of α-synuclein-rich inclusions in the surviving cell bodies. An animal model that recapitulates the main hallmarks of the disease is a powerful tool to elucidate the mechanisms at the origin of the disease and to find new treatments. Application of this model in transgenic and knockout mouse lines provides the possibility to investigate the molecular pathways leading to PD and to study the effect of other PD-related genes on α-synuclein-induced cell death. Moreover, viral vector-based animal models of PD, such as the one described here, constitute a rapid platform for the screening of new potential therapies aiming at halting or modifying the progression of this devastating neurodegenerative disorder.

## Abbreviations

αsyn+: α-synuclein-positive; μl: Microlitre; aa: Amino acid; ANOVA: Analysis of variance; d: Days; eGFP: Enhanced green fluorescent protein; GFAP: Glial fibrillary acidic protein; GAPDH: Glyceraldehyde 3-phosphate dehydrogenase; GC/ml: Genome copies per millilitre; Inj: Injected side; kDa: Kilo Dalton; LBs: Lewy bodies; NeuN: Neuronal nuclei; N-inj: Non-injected side; PBS: Phosphate-buffered saline; PD: Parkinson’s disease; P-S129 αsyn: Phosphorylated serine 129 α-synuclein; rAAV: Recombinant adeno-associated viral; SN: Substantia nigra; STR: Striatum; TBS: Tris-buffered saline; TH: Tyrosine hydroxylase; w: Weeks; WT: Wild-type.

## Competing interests

The authors declare that they have no competing interests.

## Authors’ contributions

MOS made substantial contribution to conception and design of the study, performed all *in vivo* experiments and wrote the manuscript. AVdP participated in the viral vector constructions. NC participated in the design of the western blotting study and carried out its analysis. SN participated in the analysis of the western blotting study. SS and RD participated in the design of the motor tests. CVdH participated in the viral vector constructions and productions, and in the design of the *in vivo* experiments. VB conceived and designed the study, and helped to write the manuscript. All authors read and approved the final manuscript.
